# A multiplex PCR assay for the differentiation of *Mycobacterium tuberculosis* complex reveals high rates of mixed-lineage tuberculosis infections among patients in Ghana

**DOI:** 10.3389/fcimb.2023.1125079

**Published:** 2023-04-03

**Authors:** Wellington Owusu, Arnoud H. M. van Vliet, Natalie E. Riddell, Graham Stewart, Winifred C. Akwani, Sherihane Aryeetey, Rejoice Agyeiwaa Arthur, Augustina Angelina Sylverken, Suzanne M. Hingley-Wilson

**Affiliations:** ^1^ Department of Microbial Sciences, School of Biosciences, Faculty of Health and Medical Sciences, University of Surrey, Guildford, United Kingdom; ^2^ Department of Comparative Biomedical Sciences, School of Veterinary Medicine, Faculty of Health and Medical Sciences, University of Surrey, Guildford, United Kingdom; ^3^ Department of Biochemical Sciences, School of Biosciences and Medicine, University of Surrey, Guildford, United Kingdom; ^4^ Kumasi Centre for Collaborative Research in Tropical Medicine, Kwame Nkrumah University of Science and Technology, Kumasi, Ghana; ^5^ Department of Theoretical and Applied Biology, Kwame Nkrumah University of Science and Technology, Kumasi, Ghana

**Keywords:** *Mycobacterium tuberculosis* complex, bioinformatic analyses, multiplex polymerase chain reaction, mixed-lineage tuberculosis infections, tuberculosis diagnosis, Ghana

## Abstract

In low-resource settings with high tuberculosis (TB) burdens, lack of rapid diagnostic methods for detection and differentiation of *Mycobacterium tuberculosis* complex (MTBC) is a major challenge affecting TB management. This study utilized comparative genomic analyses of MTBC lineages; *M. tuberculosis*, *M. africanum* Lineages 5/6 and *M. bovis* to identify lineage-specific genes. Primers were designed for the development of a Multiplex PCR assay which was successful in differentiating the MTBC lineages. There was no cross-reaction with other respiratory pathogens tested. Validation of the assay using clinical samples was performed with sputum DNA extracts from 341 clinically confirmed active TB patients. It was observed that 24.9% of cases were caused by *M. tuberculosis*, while *M. africanum* L5 & L6 reported 9.0% and 14.4%, respectively. *M. bovis* infection was the least frequently detected lineage with 1.8%. Also, 27.0% and 17.0% of the cases were PCR negative and unspeciated, respectively. However, mixed-lineage TB infections were recorded at a surprising 5.9%. This multiplex PCR assay will allow speciation of MTBC lineages in low-resource regions, providing rapid differentiation of TB infections to select appropriate medication at the earliest possible time point. It will also be useful in epidemiological surveillance studies providing reliable information on the prevalence of TB lineages as well as identifying difficult to treat cases of mixed-lineage tuberculosis infections.

## Introduction

Human tuberculosis (TB) is a communicable disease caused by some members of the Mycobacterium tuberculosis complex (MTBC), mainly; *Mycobacterium tuberculosis* (Mtb)*, Mycobacterium africanum* (Maf) and *Mycobacterium bovis* (Mbo). It is one of the leading causes of death from a single infectious organism, infecting about a quarter of the world’s population (WHO, 2020). It remains a global pandemic, despite the availability of interventional control measures such as the use of a live attenuated vaccine (BCG) and multi-drug therapy. The situation has been further aggravated by the lack of rapid and reliable, point-of-care diagnostic methods for low-resource areas, and the use of various forms of insufficient treatment procedures among poor resource countries (WHO, 2020).

TB in an individual is often assumed to be caused by a single clonal MTBC lineage, although mixed infections have been previously noted (Hingley-Wilson et al., 2013). Advances in molecular-based approaches in TB studies also demonstrated multiple lineages causing TB in the same patient (Van Rie et al., 2005; Huyen et al., 2012; Zetola et al., 2014) and the occurrence of mixed-lineage TB infections in high TB endemic regions has been reported (Cohen et al., 2011). In TB management, mixed-lineage TB infections have been strongly associated with poor treatment outcome (Zetola et al., 2014).

West-Africa has one of the highest incidences of TB world-wide with a unique set of circulating MTBC species namely: *M. tuberculosis, M. africanum* and *M. bovis*. While *M. tuberculosis* is generally the predominant pathogen for human TB, unusually almost 50% of all TB cases in West Africa are caused by *M. africanum* (Mostowy et al., 2004). In The Gambia, 39% of TB cases are caused by *M. africanum* (de Jong et al., 2010a). In Ghana, *M. africanum* rates remain stable at around 20%, with one of the highest rates of infections in the Northern part of Ghana (De Jong et al., 2009). While the reservoir of infection for *M. tuberculosis* is the latently infected human population, a non-human reservoir of infection for *M. africanum* in Ghana has been postulated, likely to be more concentrated in Northern Ghana (Otchere et al., 2018).

The gold standard of TB diagnosis is the isolation of MTBC by culture and the use of biochemical tests (Gholoobi et al., 2014). However, these methods are very laborious and time-consuming which further risk aggravating the condition of patients due to delayed treatment. In addition, with culturing-based techniques in a mixed infection, the fastest growing is often noted as a single infection (Hingley-Wilson et al., 2013). Species differentiation is often challenged with misidentification. For instance, *M. africanum* Lineage 5 (MafL5) and Lineage 6 (MafL6) exhibit growth characteristics which are intermediates of both *M. tuberculosis* and *M. bovis* (de Jong et al., 2010b). Since 2010, WHO recommended the use of GeneXpert assay in diagnostic facilities as a first-line TB diagnostic tool (Goig et al., 2019). It detects MTBC through the identification of insertion sequence (*IS6110)* as well as identifying rifampicin resistant genes. Although an improved modified GeneXpert Ultra version has been produced with high sensitivity and specificity, it is unable to differentiate the individual MTBC lineages to inform selection of appropriate medication.

In low-resource regions, MTBC lineages are often not differentiated prior to treatment due to reasons such as unavailability of high cost, non-portable genome sequencing machines and length of time for culture results. This can lead to inappropriate treatment regimens, for example, *M. bovis* is intrinsically resistant to pyrazinamide, one of the frontline drugs used collectively for standard TB treatment (Oryan et al., 2022). Indeed, patient exposure to prolonged pyrazinamide treatment can result in hepatotoxicity and polyarthralgia (Papastavros et al., 2002) and should therefore be avoided if not required. Additionally, antibiotic treatment duration of *M. bovis* infections is recommended for 9 months (rather than the standard 6 months) because of the absence of pyrazinamide efficacy (Lan et al., 2016). In general, TB treatment durations shorter than recommendation may lead to incomplete sterilization of an infection and increase the risk of the development of antibiotic resistance (Khalif Ali et al., 2017; Ali et al., 2019). It is therefore important to investigate and identify lineage-specific TB molecular markers for designing diagnostic assays with high level of sensitivity and specificity to inform selection of appropriate medication to limit morbidity and drug resistance.

Using the comparative genomics workflow previously described by Akwani et al., 2022, MTBC lineage-specific genes identified were transferred into the development of multiplex PCR assay for TB lineage differentiation. This will enhance precise disease diagnosis, improve epidemiological surveillance studies and help inform selection of appropriate TB drug regimens at early time point especially in low resource settings with high TB incidence.

## Materials and methods

### Selection and processing of genome sequences

**Figure 1:**
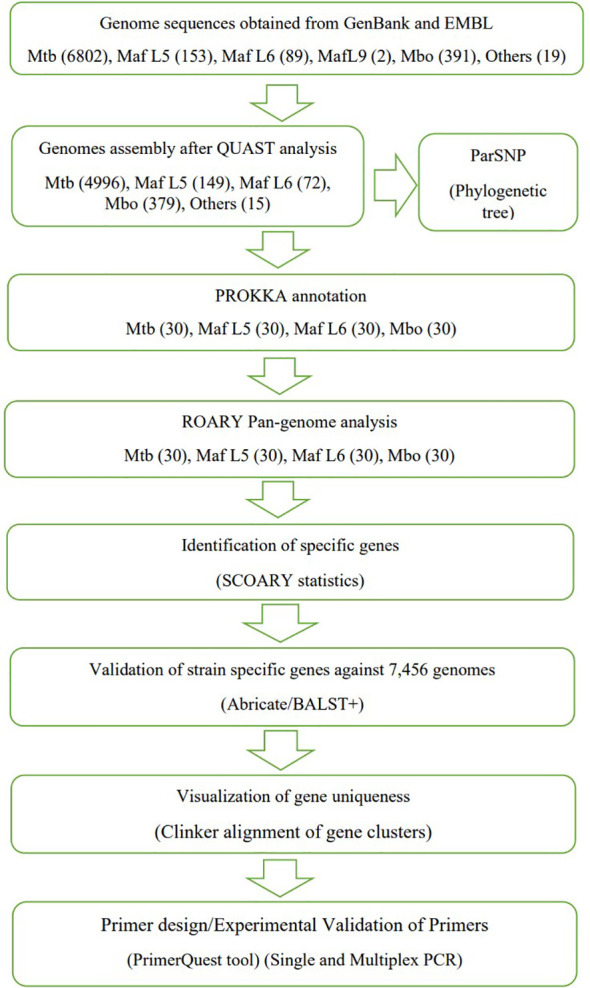
An overview of Scheme of work towards identification of lineage-specific genes for PCR primers development (similar to comparative genomic workflow previously described by Akwani et al., 2022).

Genome sequences of *M. tuberculosis, M. africanum* and *M. bovis* in the form of sequence reads and assembled genomes were obtained from NCBI, Genbank and EMBL-EBI repositories using fastq-dump instructions (SRA-Tools-NCBI, 2021). In addition, reference sequences were also obtained. An overall total of 7,456 genome sequences comprising *M. tuberculosis* (6802), *M. africanum* (244), *M. bovis* (391) and other animal-adapted MTBCs (19) were assessed (**Supplementary Table S1**). Genome assembly was performed with Shovil Megahit toolkit version 1.2.9. To evaluate the consistency of the assembled genomes, quality assessment was performed with QUAST version. 4.6.3 (Gurevich et al., 2013). The inclusion criteria for checks included: largest contig must be greater than 100kb, N50 >25kb, L50 < 50 and the genomic size between 4.0 and 4.8 Mbp. A total of 120 genomes of *M. tuberculosis*, *M. africanum* and *M. bovis* were used for the pangenome analysis leading to the identification of lineage-specific genes as shown in **Figure 1**.

### Phylogenetic analysis of MTBC

ParSNP v. 1.2 was used as described previously (Pornsukarom et al., 2018) using the “-a 13 – x” settings to generate a phylogenetic tree of MTBC lineages. Classification of lineages was achieved based on the phylogenetic tree constructed. The output was visualised with FigTree version 1.4.3.

### Comparative genomic analysis and identification of lineage-specific genes

Genomes were annotated using Prokka v1.14 (Seemann, 2014), while pangenomes were analyzed using Roary v3.12 (Page et al., 2015) at default settings and 90% BLAST cut-offusing randomly selected 120 genomes comprising *M. tuberculosis* (30), *M. africanum* L5 (30), *M. africanum* L6 (30) and *M. bovis* (30) **(** **Supplementary Table S1**). Scoary analysis (Brynildsrud et al., 2016) was used to examine the association between accessory (lineage-specific) genes and phenotypic traits. The number of lineage-specific genes was trimmed using statistical results from Bonferroni corrected p-value of ≤ 0.05. Also, lineage-specific genes were selected only if present in greater than 90% of the respective lineages and less than 10% in the other lineages. Further screening of the lineage-specific genes was performed by BLAST+ version 2.13.0 against all 7,456 MTBC genomes *via* Abricate v.1.0.9 (https://github.com/tseemann/abricate) with minimum coverage of 70% and minimum identity of 80% for a correct match. Genomic regions were compared to identify uniqueness using Clinker alignment of complete genomes (Gilchrist & Chooi, 2021).

### Isolation of genomic DNA

The following reagents were obtained through BEI Resources, NIAID, NIH: genomic DNA from *M. africanum* strains NLA009502090, NR-49655 and *M. africanum* strain NLA000017316, NR-49652. Heat-killed *M. tuberculosis* (H37Rv) and *M. bovis* (AF2122/97) were obtained from liquid cultures prepared in the containment level 3 (CL3) lab before being transferred to the CL2 lab for DNA extraction. Genomic DNA of mycobacterial strains was extracted using the cetyltrimethylammonium bromide (CTAB)-chloroform method as described previously (Belisle et al., 2009). The concentration and purity of DNA was determined by the NanoDrop 2000 at absorbance of 260nm and purity A_260_/A_280_ ratio of 1.7 to 2.0.

### Primer design

Candidate genes identified were selected for primer design, using the PrimerQuest Tool developed by Integrated DNA Technologies (https://eu.idtdna.com/Primerquest). The FASTA format of each nucleotide sequence was inputted with PCR 2 primer options. Each primer was assigned a specific product size ranging from 100 to 1000 bp. Details of primers have been shown in **Table 1**.

**Table 1:** MTBC lineage-specific genes and primer sequences.

MTBC	Gene	Type of Primer	Sequence	Length	Tm	Amplicon (bp)
Mtb	*Rv1977*	forward	GTTTCCCGAGATCAGCTCAA	20	62	418
		reverse	CATCATCATCGTGCGGTACA	20	62	
	*Rv2073c*	forward	CGCTGCTCCGGTAGTAATTT	20	62	558
		reverse	CGCCCGATGACGAATCC	17	62	
	*Rv2074*	forward	GCGATGGTCAACACCACTA	19	62	133
		reverse	GGTCGAAGGTGAAACCTACC	20	62	
Maf (L5)	*Rv3347c*	forward	CGCGGAAGCCTTAGGAAAT	19	62	275
		reverse	ACGACCCGTTTATCAGCATC	20	62	
Maf (L6)	*Rv0186 (BglS)*	forward	CCGCAACTTCGAGTACCTTT	20	62	381
		reverse	ATACCGTTGTGGTGCTTGAG	20	62	
MTB Complex	*Rv3903c* (positive control)	forward	CGGATCGAACCACCAGAATC	20	62	636
		reverse	GGCCGGATTGTCTGTAAAGT	20	62	
Mbo*	*pncA*	forward	ATGCGGGCGTTGATCATCGTC	21	62	186
		reverse	CGGTGTGCCGGAGAAGTCG	19	62	

*M.bovis primers designed from pyrazinamidase (pncA) by de los Monteros et al., 1998 were employed. The pncA gene carries a mutation within the genome of *M. bovis* but conserved in other MTBCs. There is a point mutation at the 169 nucleotide position which is occupied by guanine instead of cytosine.

### Preparation of PCR assay

For the single PCR assays, a final volume of 25 µl was setup. Each setup contained 12.5 µl of 2x GoTaq^®^ Hot Start Green Master Mix (400 µM polymerase, 400 µM of dNTPs, 4 mM MgCl_2_ and pH 8.5 buffer), produced by Promega, UK, 1µl each of 10µM forward and reverse primers, 1 µl DNA (< 250 ng), 1 µl DMSO and nuclease free water. The non-template control consisted of the master mix, specific primers and nuclease free water, while 1 µl of *E.coli* DNA was used as negative control. For the multiplex PCR assays, a 50µl reaction volume was achieved with the following constituents: 25 µl of 2x GoTaq^®^ Hot Start Green Master Mix, 5 µl of 10 µM of forward/reverse primers (1 µl of each lineage-specific primer), 1µl DNA (<250 ng), 2 µl DMSO and nuclease free water. An all-in-one multiplex PCR had 4 µl of DNA (1µl from each lineage). The reaction mix contained an excess of primers and nucleotides to ensure reaction continuity without limitation. The amplification was carried out in the SimpliAmp Thermal cycler at an initial denaturation of 2 mins at 95°C; 30 cycles of 30 sec at 95°C; 1 min at 62°C; 1 min at 72°C and a final extension at 72°C for 5 min. The separation of PCR products was performed using 2% gel agarose electrophoresis at 80 V for 1.30 hrs. A 100 bp DNA ladder was used as indicator. Visualization of gel was performed under ultraviolet light of Microtek MiBio Fluo version1.04.

### Ethical clearance

Ethical approval for the use of human sputum samples was granted by the Committee on Human Research and Publication Ethics (CHRPE) at the School of Medical Science of Kwame Nkrumah University of Science and Technology (KNUST), Ghana: (CHRPE/AP/396/22).

## Results

### Stratification and identification of lineage-specific genes of the MTBC

**Figure 2:**
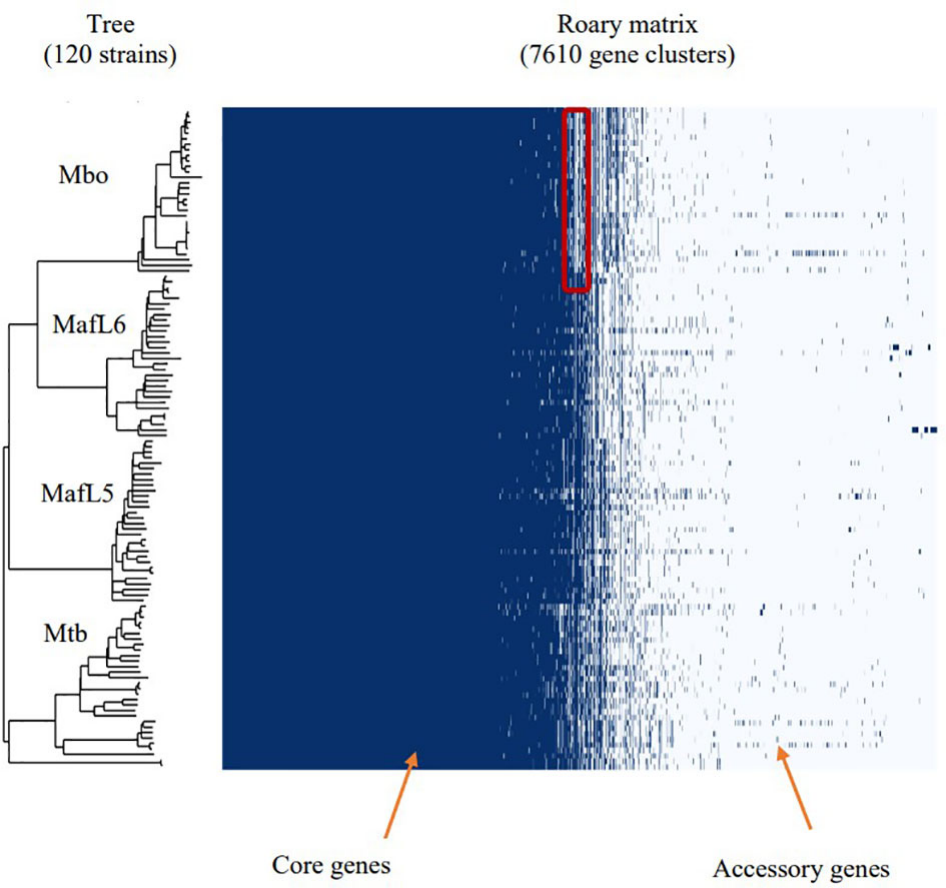
Phylogenetic tree together with pangenome distribution of genes within the MTBC – *M. tuberculosis, M. africanum* and *M. bovis*. The phylogenetic tree was constructed *via* identification of core genome SNPs using ParSNP tool. Roary matrix obtained from pangenome analysis shows the distribution of core and accessory genes within the MTBC. The highlighted section of the *M. bovis* genomes shows deletions which are characteristic of *M. bovis* as RD4, RD7, RD8, RD9 and RD12 deletions.

A selection of 120 MTBC genomes (30 *M. tuberculosis*, 30 *M. africanum* L5, 30 *M. africanum* L6 and 30 *M. bovis*) from GenBank and EMBL repositories, were subjected to comparative genomic analysis. The phylogenetic relationship between the MTBCs was established with ParSNP which constructs a phylogenetic tree using core genome SNPs. In **Figure 2**, divisions were observed in four large clusters representing *M. tuberculosis*, *M. africanum* L5, *M. africanum* L6 and *M. bovis*. Pangenome analysis was performed on the same set of genomes to obtain the distribution of gene families within the MTBCs. A Roary matrix shows the clustering of 7,610 genes into either core genes (commonly shared by all members) or accessory genes (found in only few members) (**Figure 1**). It could be seen that almost all the genes are skewed toward the core gene section while only a few were categorised as accessory genes. This type of gene distribution highlights the high level of clonality of the MTBC.

**Figure 3:**
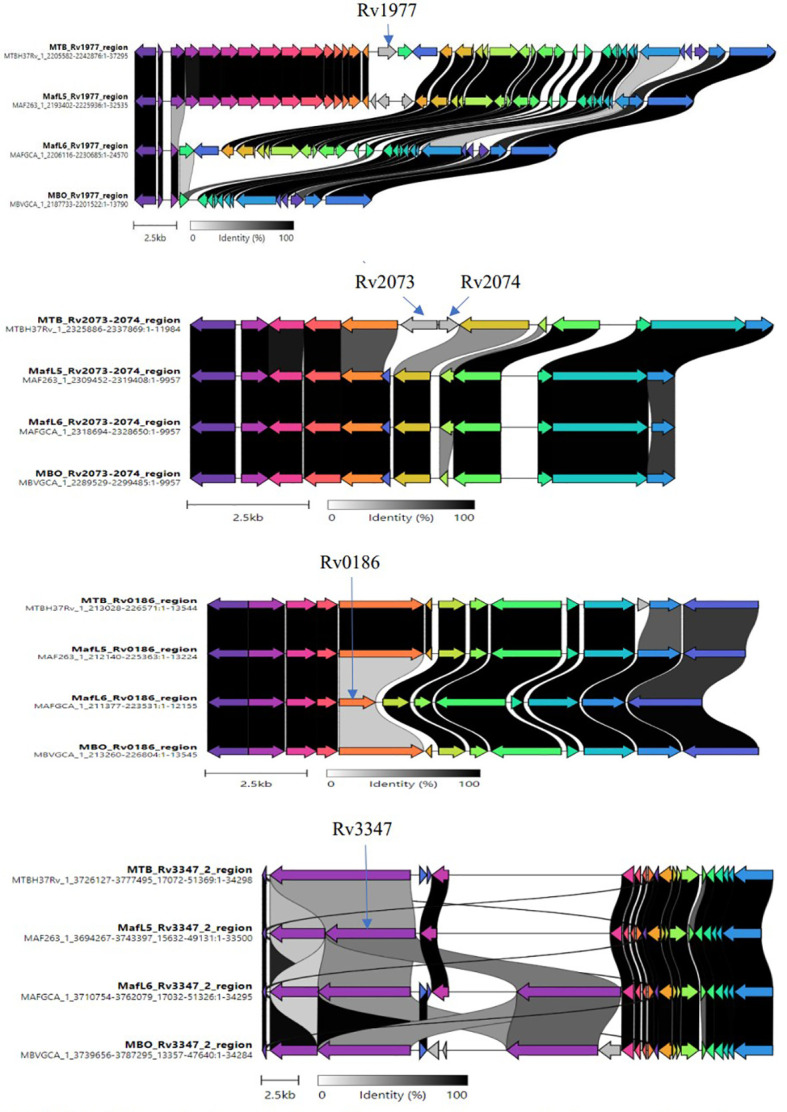
Clinker showing variations of lineage-specific genes *via* alignment of gene clusters. Variable regions of the identified unique genes were examined through the alignment of gene clusters from members of the MTBC (*M. tuberculosis, M. africanum* L5/L6*, M. bovis*).

Further analysis was conducted on the pangenome outcome to ascertain the relationship between accessory genes and trait (lineages) using Scoary statistics. The definition of lineage-specific genes was set as being present in more than 90% of specific species and less than 10% in the other lineages. A total of 56 lineage-specific genes were obtained comprising 16 *M. africanum* L5, 10 *M. africanum* L6, 10 *M. tuberculosis* and 20 *M. bovis* specific genes (**Supplementary Table S2**). A final screening of these lineage-specific genes was performed by BLAST against 7,456 MTBC genomes *via* Abricate with a minimum coverage of 70% and minimum identity of 80% for a correct match as shown in **Table 2**. The candidate genes specific for *M. tuberculosis* were *Rv1977, Rv2073c and Rv2074.* The *Rv0186*-betaglucosidase was unique for *M. africanum* L6 while *Rv3903c* was conserved in all the MTBCs, thus serving as positive control marker. The Rv*3347c* was unique for *M. africanum* L5 *via* Clinker alignment of gene clusters shown in **Figure 3**. Although BLAST hits did not show any unique gene for *M. bovis*, the *pncA* gene highlighted to be distinctive in *M. bovis* by de los Monteros et al., 1998 was used.

**Table 2:** Summary of Abricate BLAST results showing MTBC lineage-specific genes.

Gene ID	MafL5	MafL6	Mbo	Mtb	*Others	Name of gene in official H37Rv	Remarks
MAFGCA_01990	0.0	98.6	0.0	0.0	0.0	*Bgls (Rv0186)*	L6 specific
MTBH37Rv_02010	100.0	0.0	98.9	100.0	100.0		not L6
MTBH37Rv_13290	100.0	100.0	0.0	99.6	75.0		not Mbo
MTBH37Rv_13300	100.0	100.0	0.0	100.0	75.0		not Mbo
MTBH37Rv_15940	0.0	100.0	0.0	99.6	100.0		not L5/Mbo
MTBH37Rv_20850	0.7	0.0	0.0	100.0	8.3	*Rv1977*	Mtb-specific
MTBH37Rv_21880	0.7	0.0	0.0	100.0	8.3	*Rv2073c*	Mtb-specific
MTBH37Rv_21890	0.7	0.0	0.0	100.0	8.3	*Rv2074*	Mtb-specific
MTBH37Rv_41080	100.0	100.0	99.1	99.6	91.7	*Rv3903c*	positive control gene

*Others = genomes of animal-adapted ecotypes of the MTBC (M. microti, M. pennipedii, M. orygis, M. caprae, M. mungi).

### Comparison of genomic regions by clinker

The uniqueness of lineage-specific genes was visualized by comparing gene clusters *via* Clinker software as shown in **Figure 3**. Variable regions of genes were observed to aid primers design.

### Single PCR assays showing MTBC lineage-specificity

**Figure 4:**
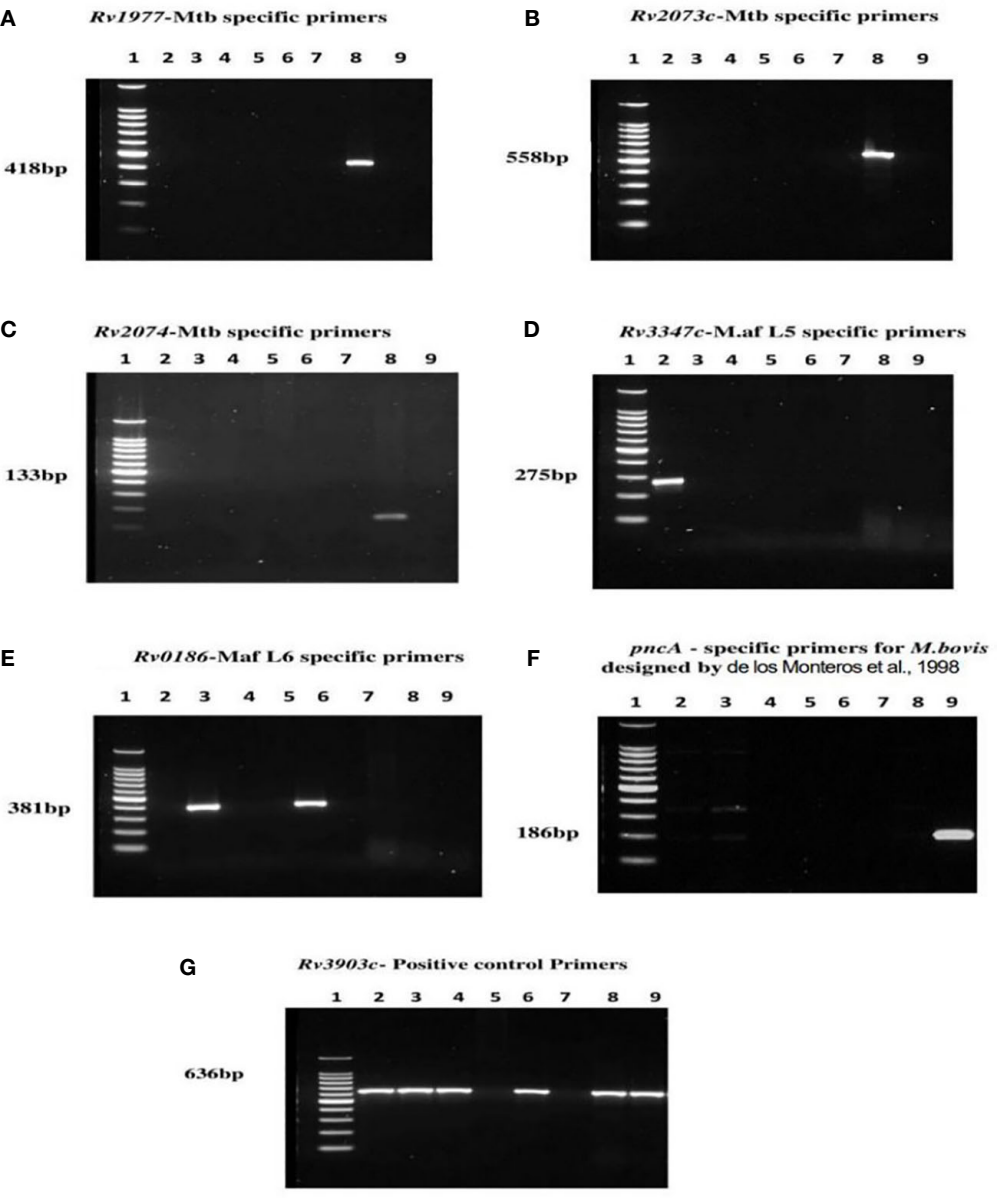
PCR results of MTBC specific lineages after optimization of various product sizes; Lane 1= DNA ladder, 2= *M. africanum* L5, 3= *M. africanum* L6 (isolate a), 4=BCG, 5= *E*. *coli*, 6= *M. africanum* L6 (isolate b), 7= water, 8= *M. tuberculosis* and 9= *M. bovis*. Each of the primers was tested against genomic DNA of all MTBCs for specificity. DNA of *E*. *coli* and nuclease-free water were used as negative and non-template controls respectively. Optimum separation of PCR products was achieved with 2% agarose gel at 80V, 1hr:30mins. Primers designed from genes; **(A)** *Rv1977*, **(B)** *Rv2073c*, and **(C)** *Rv2074* amplifying at 418 bp, 558 bp and 133 bp respectively were specific for Mtb. The *M. africanum* L5 and *M. africanum* L6 primers were set at 275 bp and 381bp respectively as shown in **(D, E)**. Primers developed from *pncA* gene by de los Monteros et al., 1998 were used for *M. bovis* identification **(F)** at 186 bp. The **(G)** *Rv3903c* primers at 636 bp served as positive control since it was conserved in all MTBCs.

The primerQuest tool was used to design and assign all primers to different PCR product sizes for the purpose of differentiating the MTBCs in a multiplex PCR assay. Primers were screened and selected on the bases of sensitivity, specificity and compatibility. The *M. tuberculosis* specific primers designed from *Rv1977, Rv2073c* and *Rv2074* produced single amplification products of 418 bp, 558 bp and 133 bp specifically in reactions with *M. tuberculosis* DNA and not with other members of the MTBC (**Figures 4A–C**). Primers to the *Rv3347c* gene unique to *M. africanum* L5 produced a product band size of 275 bp specifically in reaction with *M. africanum* L5 DNA (**Figure 4D**), while *M. africanum* L6- *BgIS* primers amplified a fragment of 381 bp specifically from *M. africanum* L6 DNA (**Figure 4E**). For *M. bovis pncA* primers designed by de los Monteros et al., 1998 were used and produced an *M. bovis*-specific amplicon of 186 bp (**Figure 4F**). The positive control primers (*Rv3903c)* were also assigned to 636 bp (**Figure 4G**).

### Multiplex PCR assay differentiating MTBC

**Figure 5:**
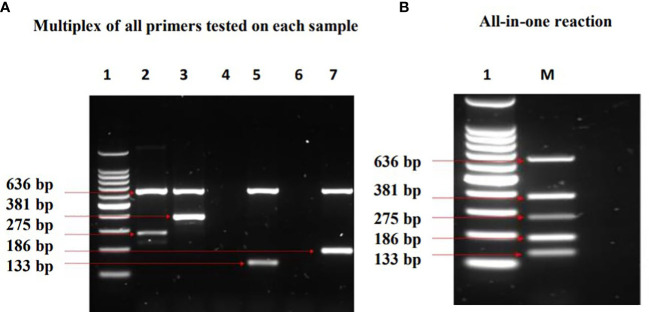
Results of Multiplex PCR assays which show identification of the MTBC lineages investigated. Lane 1= DNA ladder, 2= *M. africanum* L5, 3= *M. africanum* L6 (isolate a), 4= *E*. *coli*, 5= *M. tuberculosis*, 6= water, 7= *M. bovis*, M=mixture of all samples. In **(A)** multiplex of all primers tested on each DNA sample, two bands were observed in each of the MTBC as expected. The band at 636 bp (positive control) is conserved in all the MTBCs, whereas the other band depicts the respective lineage-specific band. Bands at 275 bp and 381 bp denote *M. africanum* L5 and L6 specificity respectively, while Mtb was represented at 133 bp. *M. bovis* was identified at 186 bp. In **(B)** All in one reaction; the compatibility and specificity of the assay was ascertained by combining all primers with mixtures of *M. tuberculosis, M. africanum* L5 & L6, and *M. bovis.* The outcome depicts a successful differentiation of the MTBCs without any inhibition.

Two forms of multiplex PCR assays were performed in a 50 µl reaction for each: Multiplex primers tested on each DNA sample (**Figure 5A**) and an “All in one” reaction i.e., combination of all primers with mixture of all DNA samples (**Figure 5B**). All expected amplification products were observed without any extra products formations.

### Limits of detection (LOD) of PCR assays

PCR experiments were performed using the identified lineage-specific primers against their respective DNA samples to identify the least amount of DNA required for amplification. *M. africanum* L5, *M. africanum* L6 and *M. bovis* recorded LOD of 0.003 ng/µl which equates to 620 genome copy numbers, while *M. tuberculosis* was detected at 0.012 ng/µl or 2479 copy numbers as shown in **Table 3**.

**Table 3:** Results showing LOD of PCR assays.

MTBC	LOD (ng/µl)	LOD (DNA copies/µl)
MafL5	0.003	620
MafL6	0.003	620
Mtb	0.012	2479
Mbo	0.003	620

A 10-fold serial dilution of DNA samples was used for the PCR to observe the least concentration at which amplification could still be achieved. DNA copy numbers were estimated using formula: (weight in ng x 6.0221x 10^23^molecules/moles)/[(genome length x 660g/mole) x 1 x 10^9^ng/g].

### Specificity of MTBC primers against other pathogens

In view of misdiagnoses of tuberculosis with other respiratory pathogens such as a range of non-tuberculous mycobacteria (NTM) (Yılmaz et al., 2017), cross-reactivity experiments involving testing primers against other microorganisms was conducted. NTMs obtained from Reference Centre for Mycobacteria, Borstel-Germany were used for the cross-reactivity study. The MTBC primers did not show any cross reactivity since negative PCR test results were obtained against all non-MTBC DNAs (**Supplementary Table S4**). Furthermore, PCR test results of other respiratory pathogens comprising a cocktail of bacteria and viruses (22 targets) also recorded negative (**Supplementary Table S5**). Details of various bacterial and viral analytes are shown in **Supplementary Table S3**.

### Validation of multiplex PCR assay using clinical samples

A total of 341 retrospective sputum samples from TB patients in Ghana were used for the validation of PCR assays. These samples have been confirmed TB positive using sputum smear microscopy, GeneXpert MTB/RIF assay and culture (BD BACTEC Mycobacterium Growth Indicator Tube- MGIT) methods based on previous studies conducted on TB drug resistance surveillance in Ghana (Sylverken et al., 2021). Sputum samples were decontaminated by treating with 4% N-Acetyl-L-Cysteine-Sodium-Hydroxide (NALC-NaOH) before neutralizing with 1X phosphate-buffered saline (PBS). DNA extraction was performed using the GenoLYSE extraction kit and followed by the multiplex PCR assay procedure described earlier. The results showed that *M. tuberculosis* contributes to a quarter (24.9%) of the cases, *M. africanum* L5 and *M. africanum* L6 were identified with 9.1% and 14.4% respectively, while *M. bovis* recorded only 1.8% of the cases. Interestingly, there was an observation of mixed-lineage TB infections at 5.9%. Also, 27.0% and 17.0% of the cases were PCR negative and unspeciated respectively, which may have been due to the extremely low concentration of DNA in some samples.

## Discussion

The ability to differentiate between the lineages of the MBTC is very important in TB management because it provides reliable information for epidemiological surveillance and treatment choice. In this study, MTBC have been phylogenetically classified leading to the identification of lineage-specific genes. These lineage-specific genes have been explored for the development of a multiplex PCR assay which distinguishes between members of the MTBC.

In low resource regions, Ziehl-Neelsen acid-fast staining microscopy is the most common technique used to diagnose TB (Denkinger et al., 2013). It requires about 5,000 – 10,000 bacilli per ml of sputum for successful detection (Ausina Ruiz et al., 2013). Thus, its limitations are low sensitivity as well as the inability to differentiate between different mycobacterial species. Although culture, biochemical tests and sequencing are considered gold standard for identification and differentiation (Gholoobi et al., 2014), these are expensive, laborious and time-consuming. Additionally, obtaining results from these methods are sometimes unreliable due to difficulty in identification of some lineages (*M. africanum* lineages exhibit growth characteristics which are intermediates of *M. tuberculosis* and *M. bovis*) (de Jong et al., 2010a). The advent of genome sequencing techniques has provided relevant data for performing extensive genomic analyses. As a result, several molecular-based assays have been designed to detect MTBCs. These methods are highly sensitive and specific because unique gene sequences are targeted for amplification. Researchers have discovered gene markers such as IS*6110, hsp65, dnaJ, psbA, lepA* and *MPT64* to detect MTBCs against other respiratory pathogens such NTMs (Chin et al., 2018). A recent multiplex PCR assay (Akwani et al., 2022) demonstrated successful separation of *Mycobacterium abscessus* complex subspecies from other NTMs as well as *M. tuberculosis*, although evaluation of assay performance in clinical samples needs to be carried out. Since 2010, WHO has recommended the use of GeneXpert MTB/RIF assay as the first-line diagnostic tool which detects MTBC together with rifampicin resistance (Goig et al., 2019). This is a molecular approach based on detection of the repetitive elements *IS6110* and IS*1081* and rifampicin resistance region. However, misdiagnoses of TB using the GeneXpert assay have been observed in NTM species at a high bacterial load (Pang et al., 2017). In TB endemic areas with infections caused by a diversity of MTBC species, a suitable differential diagnostic approach will be required since GeneXpert lacks the ability to distinguish between MTBC lineages.

In West Africa, MTBC classification has been achieved using spoligotyping technique which involves the amplification of direct repeat copies, followed by hybridization into intergenic spacers experiments (De Jong et al., 2009; de Jong et al., 2010b; Ofori-Anyinam et al., 2016; Otchere et al., 2018; Otchere et al., 2019). This is a two-step approach which is expensive, laborious and time-consuming. In Ghana, a single multiplex PCR experiment was conducted on the MTBC differentiation using primers from spacer regions 33 and 34 of the DR copies of MTBC, *IS6110* and the *hsp65* (Yeboah-Manu et al., 2001). Although this assay is not successful in separation of *M. tuberculosis* from *M. africanum* L6, the assay could still be used to complement biochemical testing.

However, the present study introduces a successful differentiation of MTBCs *via* a single multiplex PCR method which is rapid, cost effective and has a short turnaround time. The different PCR product sizes can be easily used to distinguish between lineages without the need for sequencing. One advantage of this PCR assay is the ease of adapting it to the available hardware as it will work on any PCR platform. This new assay provides a reliable solution to misdiagnoses with other NTM infections reported in some endemic regions (Brown-Elliott et al., 2012; Yılmaz et al., 2017; He et al., 2022). Indeed, our assay did not cross-react with a range of NTMs, respiratory bacterial and viral pathogens (**Supplementary Tables S4**, **S5**). We tested our assay using clinical samples (**Table 4**; **Supplementary Figure S1**) to demonstrate its utility at revealing the diversity of MTBC lineages in Ghana. The highest number of cases (24.9%) was caused by *M. tuberculosis*, followed by *M. africanum* L5 & L6 (23.5%). *M. bovis* recorded 1.8% which is comparable to 1.5% observed by Otchere et al., 2019. Negative PCR results (27.0%) and unspeciated lineages (17.0%) may require further confirmation *via* genome sequencing, although samples have been previously detected as MTBCs by liquid cultures, followed by confirmations using purity tests (on blood agar) and rapid test kit (TB cID) (Sylverken et al., 2021). However, since these are retrospective samples stored over time, sample integrity may have been compromised through repeated freeze/thaw cycles which were beyond our control. Following the reports of mixed MTBC infections among high TB burden settings (Van Rie et al., 2005; Huyen et al., 2012; Zetola et al., 2014), this study detected 20 (5.9%) cases of mixed-lineage TB infections. Poor treatment outcomes have been strongly associated with mixed-lineage TB infections (Zetola et al., 2014). Therefore, the effect of mixed-lineage TB infections in TB management cannot be overlooked as treatment failures are often observed in various regions of Ghana (Agyare et al., 2021).

**Table 4:** Results of multiplex PCR validation using samples from 341 active TB patients in Ghana.

Target	Number	Percentage (%)
*M. tuberculosis*	85	24.9
*M. bovis*	6	1.8
*M. africanum* L5	31	9.1
*M. africanum* L6	49	14.4
*M. africanum* L5*/M. tuberculosis*	6	1.8
*M. africanum* L5*/M. africanum* L6	5	1.5
*M. africanum* L6*/M. tuberculosis*	5	1.5
*M. bovis/M. africanum* L6	2	0.6
*M. bovis/M. tuberculosis*	2	0.6
Unspeciated species (only positive for control marker)	58	17.0
MTBC negative	92	27.0
TOTAL	341	100

In summary, this assay is not an alternate replacement for GeneXpert which is currently the first-line TB diagnostic tool recommended by WHO. However, it will be beneficial to low-resource regions where TB is caused by diverse members of the MTBC providing rapid diagnosis to inform appropriate TB drug selection, reduce treatment relapse and the development of antimicrobial resistance. It will also be useful in epidemiological surveillance studies providing reliable information on TB lineage prevalence as well as identifying cases of mixed-lineage tuberculosis infections.

## Data availability statement

The original contributions presented in the study are included in the article/**Supplementary Material**. Further inquiries can be directed to the corresponding authors.

## Author contributions

The concept and study design were established by SH-W, AS, NR, and GS. Bioinformatic analyses was performed by AvV. Assay optimization by WO, WA, and SH-W. Assay validation using clinical samples was performed in Ghana by RA, SA, and AS. Manuscript writing and editing were done by WO, AS, AvV, GS, and SH-W. All authors contributed to the article and approved the submitted version.
