# Supplementary material for: A multiplex PCR assay for the differentiation of Mycobacterium tuberculosis complex reveals high rates of mixed-lineage tuberculosis infections among patients in Ghana
Source: Front Cell Infect Microbiol. 2023 Apr 3;13:1125079. doi: 10.3389/fcimb.2023.1125079 (PMC10108843; doi:10.3389/fcimb.2023.1125079)
Supplement: Supplementary file 1 [file DataSheet_1.pdf]

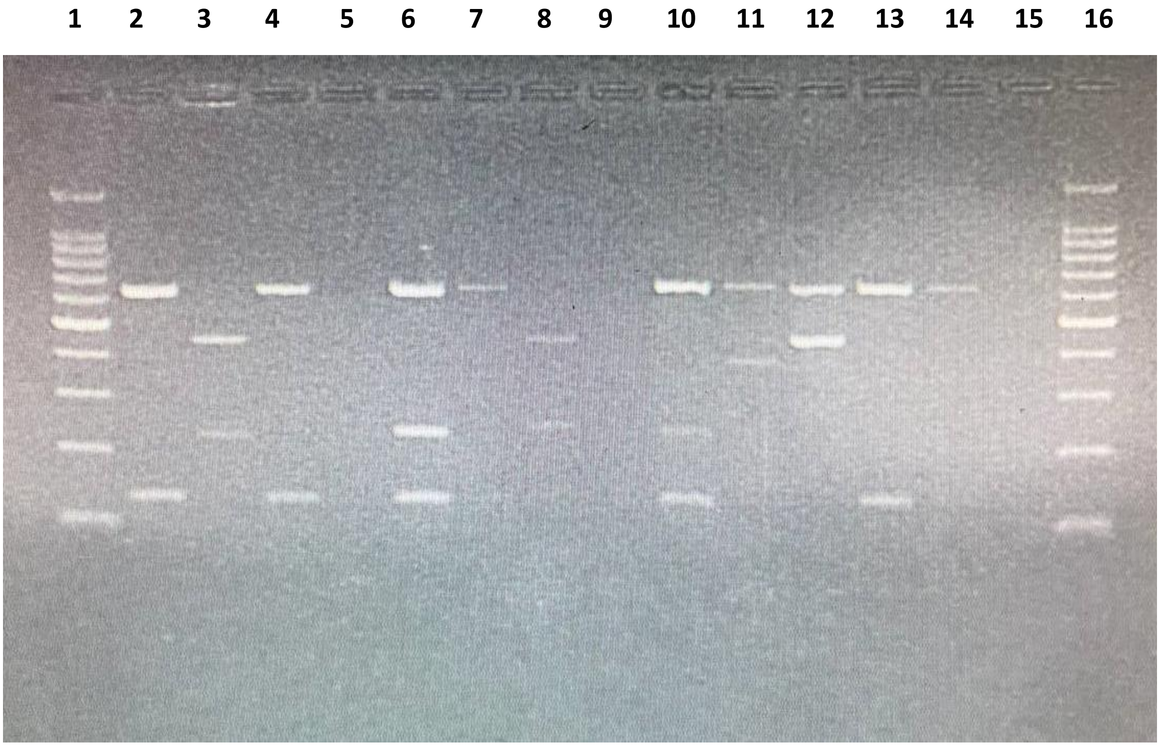

Supplementary Figure S1: Observation of Multiplex PCR assay performance using MTBC DNA derived from clinical TB sputum samples reveals lineage specificity as well as mixed-lineage infections. Optimum separation of PCR products was achieved with 2% agarose gel at 80V for 1hr:30mins. Lanes 1 and 16 = DNA ladder, 2= *M. tuberculosis* only, 3= *M. africanum* L5 and *M. africanum* L6, 4= *M. tuberculosis* only, 5= Negative sample (no DNA), 6= *M. africanum* L5 and *M. tuberculosis*, 7= Unspeciated sample, 8= *M. africanum* L5 and *M. africanum* L6, 9= Negative sample (no DNA), 10= *M. tuberculosis* and *M. africanum* L5, 11= *M. africanum* L6 only, 12= *M. africanum* L6 only, 13= *M. tuberculosis* only, 14= Unspeciated sample, 15= Negative sample (no DNA).
