# Supplementary material for: A multiplex PCR assay for the differentiation of Mycobacterium tuberculosis complex reveals high rates of mixed-lineage tuberculosis infections among patients in Ghana
Source: Front Cell Infect Microbiol. 2023 Apr 3;13:1125079. doi: 10.3389/fcimb.2023.1125079 (PMC10108843; doi:10.3389/fcimb.2023.1125079)
Supplement: Supplementary file 3 [file Table_2.docx]

**Supplementary Table S2:**

**Table S2.1: Scoary analyses with Microsoft Excel screening of *Mycobacterium africanum* L5 candidate genes**

| Gene | Annotation | MafL5  (> 90%) | MafL6/Mtb/Mbo (< 10%) |
| --- | --- | --- | --- |
| group_1645 | TetR/AcrR family transcriptional regulator | 100 | 2 |
| group_1181 | hypothetical protein | 100 | 1 |
| group_1599 | putative fatty-acid-CoA ligase fadD4 | 93 | 2 |
| group_1684 | VWA domain-containing protein | 100 | 0 |
| group_3018 | carboxymuconolactone decarboxylase family protein | 100 | 0 |
| group_960 | sensor histidine kinase | 100 | 1 |
| group_1668 | oxidase | 100 | 0 |
| group_675 | hypothetical protein | 93 | 3 |
| group_3287 | hypothetical protein | 100 | 0 |
| group_3113 | hypothetical protein | 100 | 0 |
| group_1323 | hypothetical protein | 93 | 2 |
| group_1051 | cation transporter ATPase J | 93 | 0 |
| group_2954 | hypothetical protein | 100 | 0 |
| group_1759 | PE family protein | 100 | 7 |
| bioF2_1 | putative 8-amino-7-oxononanoate synthase bioF2 | 93 | 8 |
| group_138 | PPE family protein | 93 | 9 |

**Table S2.2: Scoary analyses with Microsoft Excel screening of *Mycobacterium africanum* L6 candidate genes**

| Gene | Annotation | MafL6 (>90%) | MafL5/Mtb/Mbo (<10%) |
| --- | --- | --- | --- |
| group_3643 | beta-glucosidase BglS | 97 | 0 |
| group_1806 | ABC transporter substrate-binding protein | 93 | 3 |
| group_1804 | 5-oxoprolinase | 93 | 2 |
| group_806 | NarK family nitrate/nitrite MFS transporter | 93 | 0 |
| group_770 | PPE family protein | 93 | 2 |
| group_900 | integral membrane protein | 100 | 3 |
| group_3653 | transcription termination factor Rho | 100 | 0 |
| group_1532 | chaperone protein HtpG | 100 | 0 |
| PPE45_1 | PPE family protein | 97 | 1 |
| group_729 | glycosidase | 100 | 7 |

**Table S2.3: Scoary analyses with Microsoft Excel screening of *Mycobacterium tuberculosis* candidate genes**

| Gene | Annotation | Mtb  (> 90%) | MafL5/MafL6/Mbo  (< 10%) |
| --- | --- | --- | --- |
| group_2116 | hypothetical protein | 93 | 1 |
| group_3522 | oxidoreductase | 100 | 1 |
| group_3521 | F420-dependent biliverdin reductase | 100 | 1 |
| group_4322 | hypothetical protein | 93 | 0 |
| group_615 | galactose-1-phosphate uridylyltransferase | 90 | 3 |
| menE | long-chain fatty acid--CoA ligase | 93 | 3 |
| group_440 | fumarate reductase membrane anchor subunit | 93 | 3 |
| group_1064 | hypothetical protein | 100 | 1 |
| group_877 | proline hydroxylase | 90 | 1 |
| group_2880 | hypothetical protein | 93 | 1 |

**Table S2.4: Scoary analyses with Microsoft Excel screening of *Mycobacterium bovis* candidate genes**

| Gene | Annotation | Mbo (>90%) | MafL5/MafL6/Mtb (<10%) |
| --- | --- | --- | --- |
| pknDa | Transmembrane serine/threonine-protein kinase D pknDa | 97 | 0 |
| pstBa | Phosphate-transport protein ABC transporter pstBa | 97 | 3 |
| group_1899 | phosphate ABC transporter permease PstA | 97 | 3 |
| group_4200 | transposase | 93 | 0 |
| group_1055 | hypothetical protein | 93 | 1 |
| treZb | malto-oligosyltrehalose trehalohydrolase | 93 | 2 |
| PPE56 | PPE domain-containing protein | 90 | 1 |
| group_661 | polyketide synthase | 100 | 2 |
| group_1078 | putative oxidoreductase | 100 | 2 |
| group_1463 | oxidoreductase | 100 | 1 |
| kce_1 | 3-keto-5-aminohexanoate cleavage enzyme | 100 | 0 |
| group_4196 | hypothetical protein | 100 | 0 |
| group_588 | hypothetical protein | 100 | 4 |
| group_2933 | transposase | 100 | 0 |
| group_1120 | hypothetical protein | 100 | 0 |
| group_815 | PPE family protein PPE33 | 97 | 2 |
| group_4198 | hypothetical protein | 93 | 0 |
| group_3183 | hypothetical protein | 93 | 1 |
| group_1825 | hypothetical protein | 100 | 7 |
| group_725 | ATP-binding protein | 100 | 8 |
