# Supplementary material for: A multiplex PCR assay for the differentiation of Mycobacterium tuberculosis complex reveals high rates of mixed-lineage tuberculosis infections among patients in Ghana
Source: Front Cell Infect Microbiol. 2023 Apr 3;13:1125079. doi: 10.3389/fcimb.2023.1125079 (PMC10108843; doi:10.3389/fcimb.2023.1125079)
Supplement: Supplementary file 4 [file Table_3.docx]

**Supplementary Table S3:**

**Respiratory Control Panel (22 targets) obtained from AB-Scientific, Microbiologics**

| **Viral Analytes** | **Bacterial Analytes** |
| --- | --- |
| *Adenovirus Type 6* | *Bordetella parapertussis* |
| *Influenza A subtype H1-2009 A/California/04-2009* | *Bordetella pertussis* |
| *Coronavirus 229E* | *Chlamydophila pneumoniae CWL-029* |
| *Influenza A subtype H3 A/Wuhan/359/95* | *Mycoplasma pneumoniae* |
| *Coronavirus HKU1 surrogate* |  |
| *Influenza B/Brisbane* |  |
| *Coronavirus NL63 surrogate* |  |
| *Parainfluenza Virus 1* |  |
| *Coronavirus OC43 surrogate* |  |
| *Parainfluenza Virus 2* |  |
| *Human Metapneumovirus surrogate* |  |
| *Parainfluenza Virus 3-C243* |  |
| *Rhinovirus 1B* |  |
| *Parainfluenza Virus 4a surrogate* |  |
| *Influenza A subtype H1N1 A/New Caledonia/20/99* |  |
| *Respiratory Syncytial Virus A2* |  |
| *Influenza A subtype H3N2 A/Texas/1/1977* |  |
| *SARS-CoV-2/USA/WA1/2020* |  |
