# Supplementary material for: A multiplex PCR assay for the differentiation of Mycobacterium tuberculosis complex reveals high rates of mixed-lineage tuberculosis infections among patients in Ghana
Source: Front Cell Infect Microbiol. 2023 Apr 3;13:1125079. doi: 10.3389/fcimb.2023.1125079 (PMC10108843; doi:10.3389/fcimb.2023.1125079)
Supplement: Supplementary file 5 [file Table_4.docx]

**Supplementary Table S4: Specificity of MTBC primers against non-tuberculous mycobacterial species**

| NTM species | L5  *(Rv3347c)* | L6  *(Rv0186)* | Mtb *(Rv2074)* | Mbo  *(pncA)* | Pos.Control  *(Rv3903c)* |
| --- | --- | --- | --- | --- | --- |
| *M. abscessus subsp. abscessus*  (rough) | - | - | - | - | - |
| *M. abscessus subsp. abscessus (*smooth*)* | - | - | - | - | - |
| *M. abscessus subsp. bolleti* | - | - | - | - | - |
| *M. abscessus subsp. massiliense* | - | - | - | - | - |
| *M. avium* | - | - | - | - | - |
| *M. intracellulare* | - | - | - | - | - |
| *M. fortuitum* | - | - | - | - | - |
| *M. kansasii* | - | - | - | - | - |
| *M. chelonae* | - | - | - | - | - |
| *M. chimaera* | - | - | - | - | - |

Negative PCR result = -
