# Supplementary material for: A multiplex PCR assay for the differentiation of Mycobacterium tuberculosis complex reveals high rates of mixed-lineage tuberculosis infections among patients in Ghana
Source: Front Cell Infect Microbiol. 2023 Apr 3;13:1125079. doi: 10.3389/fcimb.2023.1125079 (PMC10108843; doi:10.3389/fcimb.2023.1125079)
Supplement: Supplementary file 6 [file Table_5.docx]

**Supplementary Table S5: Specificity of MTBC primers against other respiratory pathogens**

| Sample | L5-*Rv3347c* | L6-*Rv0186* | Mtb-*Rv2074* | Mbo-*pncA* | Pos. Control-*Rv3903c* |
| --- | --- | --- | --- | --- | --- |
| Panel 22* | Negative | Negative | Negative | Negative | Negative |

*****The respiratory control panel comprises a total of 22 targets of bacterial and viral analytes (Supplementary Table S3**).** Lyophilised product from manufacturer was dissolved in 300ul nuclease free water and treated as sample in the PCR experiment.
